# Supplementary material for: The interactome of intact mitochondria by cross-linking mass spectrometry provides evidence for coexisting respiratory supercomplexes
Source: Mol Cell Proteomics. 2017 Dec 8;17(2):216–32. doi: 10.1074/mcp.RA117.000470 (PMC5795388; doi:10.1074/mcp.RA117.000470)
Supplement: Supplemental Data [file supp_RA117.000470_133922_0_supp_23978_fzffwf.zip › spectra_annotation/mito_spectra_annotation/229-1-8-1-7-1.pdf]

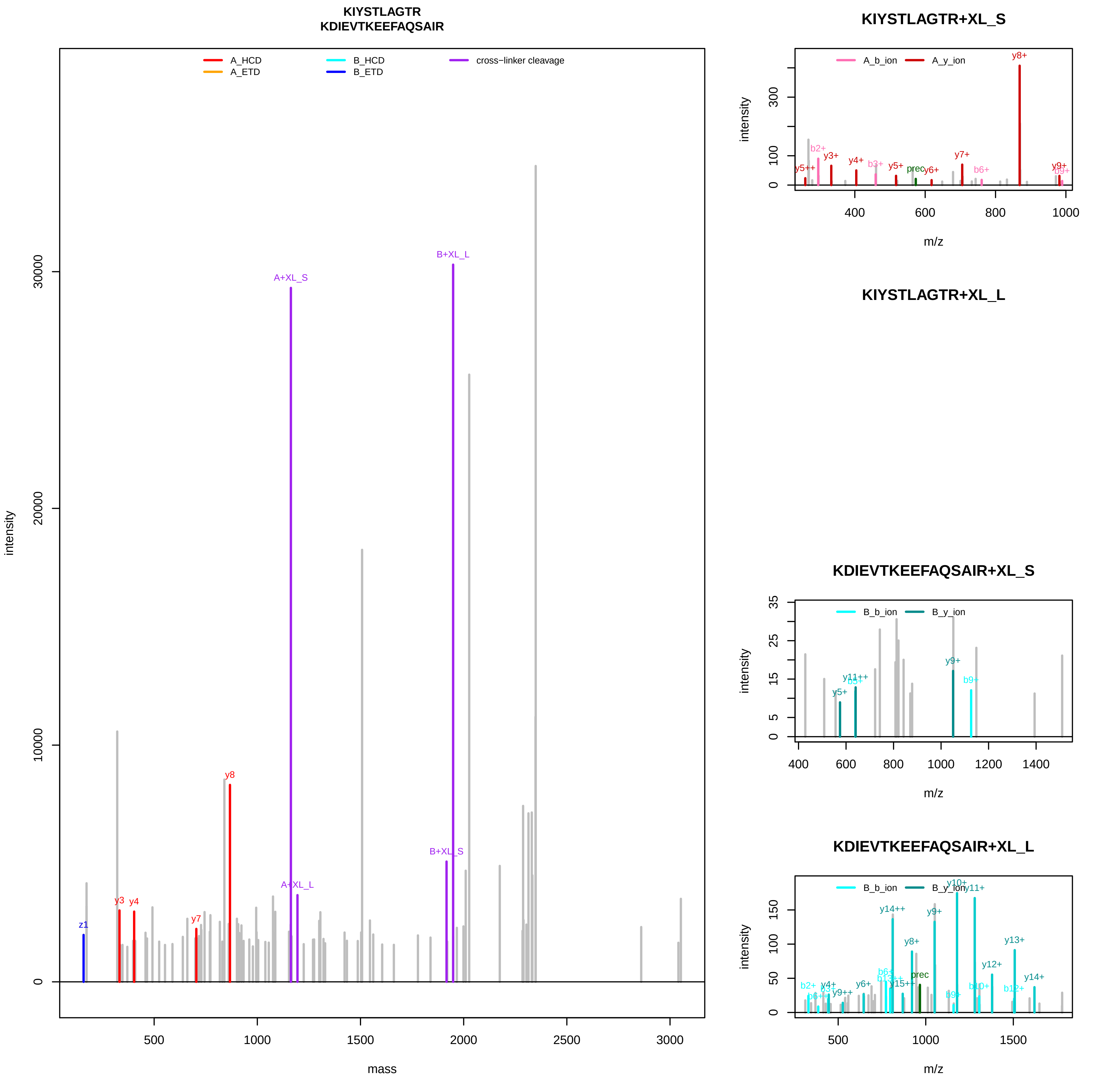
KIYSTLAGTR
KDIEVTKEEFAQSAIR
A_HCD
A_ETD
B_HCD
B_ETD
cross−linker cleavage
A+XL_S
B+XL_L
A+XL_L
B+XL_S
z1
y3
y4
y7
y8
intensity
mass
KIYSTLAGTR+XL_S
A_b_ion
A_y_ion
y5++
b2+
y3+
y4+
b3+
y5+
prec
y6+
y7+
b6+
y8+
y9+
b9+
m/z
KIYSTLAGTR+XL_L
KDIEVTKEEFAQSAIR+XL_S
B_b_ion
B_y_ion
y5+
y11++
b5+
y9+
b9+
KDIEVTKEEFAQSAIR+XL_L
y14++
y10+
y11+
y9+
y8+
y13+
y12+
y14+
b6+
y6+
y4+
b2+
prec
b10+
b12+
